# Occupational cholangiocarcinoma caused by exposure to 1,2‐dichloropropane and/or dichloromethane

**DOI:** 10.1002/ags3.12051

**Published:** 2017-11-17

**Authors:** Shoji Kubo, Shigekazu Takemura, Shogo Tanaka, Hiroji Shinkawa, Masahiko Kinoshita, Genya Hamano, Tokuji Ito, Masaki Koda, Takanori Aota

**Affiliations:** ^1^ Department of Hepato‐Biliary‐Pancreatic Surgery Osaka City University Graduate School of Medicine Osaka Japan

**Keywords:** 1,2‐dichloropropane, biliary intraepithelial neoplasia, cholangiocarcinoma, dichloromethane, intraductal papillary neoplasm of the bile duct

## Abstract

A cluster of cholangiocarcinoma among printing company workers who were exposed to 1,2‐dichloropropane and/or dichloromethane was classified by the Ministry of Health, Labour and Welfare of Japan on 1 October 2013 as “occupational cholangiocarcinoma”. At the time of the diagnosis of cholangiocarcinoma, levels of γ‐glutamyl transferase, and aspartate and alanine aminotransferases were elevated, and had been elevated in some patients several years prior to the diagnosis. Regional dilatation of intrahepatic bile ducts without tumor‐induced obstruction was characteristic in diagnostic imaging. Pathological examination found chronic bile duct injury with DNA damage, precancerous/preinvasive lesions such as biliary intraepithelial neoplasia and intraductal papillary neoplasm of the bile duct in various sites of the large bile ducts, and invasive cholangiocarcinoma such as mass‐forming type and intraductal growth‐type intrahepatic cholangiocarcinoma and mainly papillary‐type extrahepatic cholangiocarcinoma. Whole‐exome analysis of the cancerous tissues showed hypermutation, substantial strand bias, and unique trinucleotide mutational changes. Patients seemed to suffer high incidence of postoperative complications including intra‐abdominal, which might be related to chronic bile duct injury. Postoperative recurrence from multicentric origins occurred in some patients, as DNA‐injured bile ducts have high carcinogenic potential. Aggressive treatment, including second resections for such multicentric recurrences, appeared to be effective. In 2014, the International Agency for Research on Cancer classified 1,2‐dichloropropane as Group 1 (carcinogenic to humans) and dichloromethane as Group 2A (probably carcinogenic to humans) carcinogens.

## 1 INTRODUCTION

A cluster of cholangiocarcinoma (CCA) among young adult workers at the department of offset proof‐printing in a printing company in Osaka was recently reported.^1^, ^2^ Long‐term exposure to high concentrations of dichloromethane (DCM) and/or 1,2‐dichloropropane (DCP) is strongly suspected to be the cause.^1^, ^2^, ^3^ On 1 October 2013, The Ministry of Health, Labour and Welfare of Japan classified such CCA as “occupational cholangiocarcinoma”.^3^ Currently, 38 patients, some of whom were described in previous reports, are recognized as having occupational CCA.^1^, ^2^, ^4^ In the present study, we review the epidemiology, clinicopathological and molecular biological findings, treatments and outcome of occupational CCA, and discuss the mechanism of carcinogenesis.

## 2 EPIDEMIOLOGY

Cholangiocarcinoma arises from the biliary epithelium of the liver or in the extrahepatic bile ducts. Risk factors for CCA include primary sclerosing cholangitis (PSC), pancreaticobiliary maljunction, hepatolithiasis, liver flukes such as *Opisthorchis viverrini*, and chemical carcinogens such as nitrosamines. Other reported factors include inflammatory bowel disease, and hepatitis B and C virus. The International Agency for Research on Cancer (IARC) also reported aflatoxin, plutonium and thorotrast to be risk factors for CCA. Recently, a CCA cluster was reported among young adults who worked at a printing company in Osaka.^1^, ^2^ CCA was diagnosed in 18 former or current workers between November 1996 and March 2015. In the department, various chemicals, including chlorinated organic solvents such as 1,1,1‐trichloroethane (TCE), DCM, and DCP were used to clean ink residues. TCE was used until December 1992, DCM was used until March 1996, and DCP was used until October 2006. [Correction added on 8 January 2018, after first online publication: The end dates of both DCM and DCP usage have been corrected.] This department was estimated to have employed 111 former or current workers (88 men and 23 women) between 1981 and 2012. Most workers were relatively young. Up to now, CCA developed in 18 of the 111 workers. All 18 of these patients were exposed to DCP, and 12 were exposed to DCM. When CCA was diagnosed in 17 patients (as of December 2012), the estimated overall standardized incidence rate (SIR) was 1130 (95% confidence interval: 660–810).^5^ In addition, CCA incidence increased with cumulative exposure to DCP, which implies an exposure‐response relationship. Notably, for cumulative exposure more than 1000 ppm‐year, SIR was more than 10 000.^6^

Two workers in this company had gastric cancer, one had Bowen's disease, and one had renal carcinoma. Another worker with trichloroethylene exposure developed severe acute hepatitis.^7^

Currently, 38 patients are recognized to have occupational CCA; each was exposed to high concentrations of DCP and/or DCP for at least 3 years.^8^, ^9^, ^10^ These patients had no other known risk factors for CCA (such as PSC, hepatolithiasis, pancreaticobiliary maljunction, or liver fluke infection).

## 3 CLINICAL FINDINGS, LABORATORY TEST RESULTS AND DIAGNOSTIC IMAGING

The 18 patients from the printing company in Osaka ranged in age from 25 to 48 years old. Their periods of exposure to the chemicals prior to diagnosis of CCA ranged from 6 years, 1 month to 16 years, 1 months (median: 12 years, 6 months). While working at the printing company, some workers suffered from headache, nausea, vomiting, dermatitis, and/or enhanced flare after drinking. CCA was diagnosed in five of the 18 patients when they presented with abdominal pain, jaundice, and/or appetite loss; in 11 patients who had abnormal liver function test results or liver tumors detected during regular health examinations; and in two patients whose liver dysfunction was detected during treatment for other diseases.

At the time of diagnosis, serum γ‐glutamyl transferase (γ‐GGT) activity was elevated in all patients, serum activities of aspartate aminotransferase (AST) and alanine aminotransferase (ALT) and serum concentrations of total bilirubin were elevated in most patients. In some patients, serum γ‐GGT activity gradually increased for several years prior to their CCA diagnosis, followed by increased activities of AST and ALT.^11^, ^12^ Such increased γ‐GGT activity must be related to chronic bile duct injury by DCP and/or DCM. Serum concentrations of carcinoembryonic antigen (CEA) and carbohydrate antigen 19‐9 (CA 19‐9) were elevated in 11 and 13 of the 18 patients, respectively.

**Figure 1:**
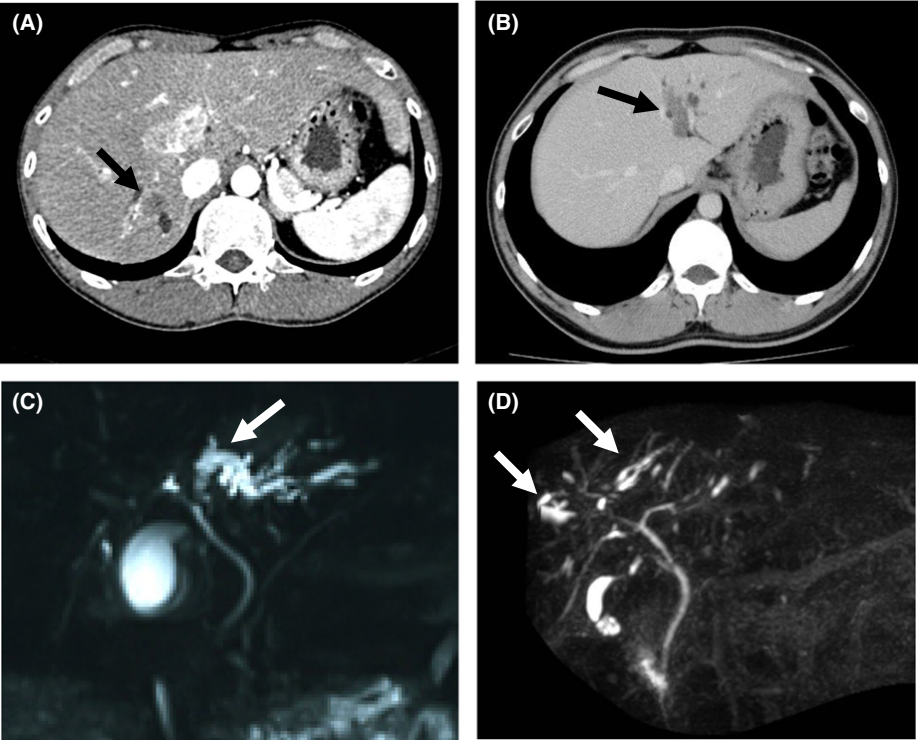
Diagnostic imaging of patients with occupational cholangiocarcinoma.^2^ (A) Mass‐forming type of intrahepatic cholangiocarcinoma (arrow). (B) Intraductal growth type of intrahepatic cholangiocarcinoma (arrow). (C) Intrahepatic bile ducts dilated with cholangiocarcinoma‐induced obstruction of the bile ducts (arrow). (D) Intrahepatic bile ducts, dilated but without tumor‐induced obstruction (arrows)

**Figure 2:**
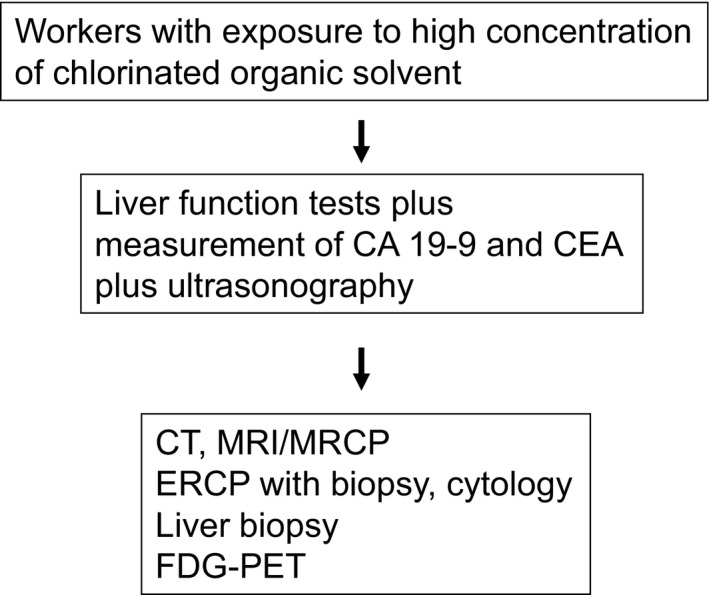
Screening and surveillance of cholangiocarcinoma in workers exposed to chlorinated organic solvents.^12^ CEA, carcinoembryonic antigen; CT, computed tomography; ERCP, endoscopic retrograde cholangiopancreatography; FDG‐PET, fluorodeoxyglucose–positron emission tomography; MRCP, magnetic resonance cholangiopancreatography; MRI, magnetic resonance imaging

Diagnostic imaging (including computed tomography [CT], magnetic resonance imaging [MRI], magnetic resonance cholangiopancreatography [MRCP], ultrasonography and endoscopic retrograde cholangiopancreatography [ERCP] showed space‐occupying lesions, bile ducts with papillary, villous, or protruding tumors, stenosis or obstructions of the bile ducts and dilation of peripheral sites of the bile duct, and dilated intrahepatic bile ducts without tumor‐induced obstruction (Figure 1). Dilated intrahepatic bile ducts without tumor‐induced obstruction were characteristic of occupational CCA and apparently corresponded to findings of PSC, including multiple intrahepatic bile duct strictures, with or without fusiform dilatation.^13^ Ultrasonography detected the occupational CCA in all 18 patients, which indicates that regular health examinations with a combination of ultrasonography and laboratory tests (including γ‐GGT, AST, ALT, CA19‐9, and CEA levels) can help screen for occupational CCA.^12^ CT, MRI, and MRCP can detect mass lesion and characteristic bile duct findings. Definitive diagnosis requires liver biopsy and biopsy during ERCP (Figure 2).

Studies by our department^14^ and of patients with sporadic young‐onset CCA in the Rosai Hospital Group database^15^ showed occupational CCA patients had a higher percentage of regional bile duct dilatation than did patients with non‐occupational CCA.

Similar clinical findings, laboratory test results and diagnostic imaging findings were observed in most patients with occupational CCA in Japan.^4^

## 4 PATHOLOGICAL FINDINGS

**Figure 3:**
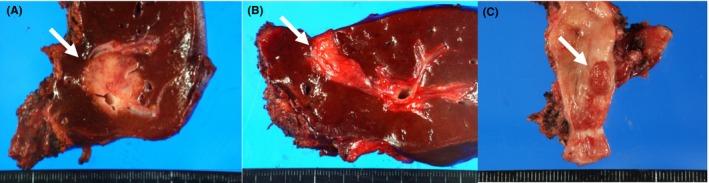
Surgical specimens. (A) Mass‐forming type of intrahepatic cholangiocarcinoma (arrow).^2^ (B) Intraductal growth type of intrahepatic cholangiocarcinoma (arrow).^2^ (C) Extrahepatic cholangiocarcinoma of the papillary type (arrow)

**Figure 4:**
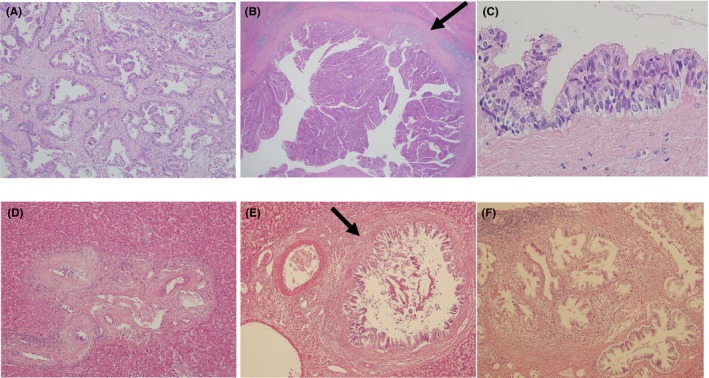
Pathological features of surgical specimens in patients with occupational cholangiocarcinoma. (A) Mass‐forming type of intrahepatic cholangiocarcinoma with tubular adenocarcinoma (H‐E staining, ×400).^2^ (B) Intraductal growth type of intrahepatic cholangiocarcinoma with focal invasion (arrow, ×150).^2^ (C) Biliary intraepithelial neoplasia‐3 (×300).^2^ (D) Sclerotic lesion in intrahepatic bile duct (×300). (E) Biliary epithelial hyperplasia (arrow). (F) Hyperplasia of the peribiliary glands (×300)

**Figure 5:**
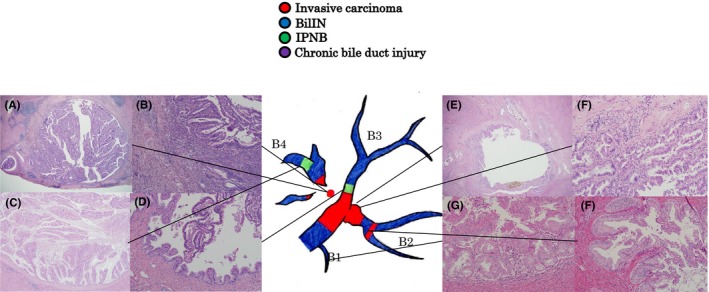
Pathological findings corresponding to bile duct imaging: mapping chart of atypical epithelium (H‐E staining).^16^ (A) (×40), (B) (×100) The main lesion in segment 4 (B4) is a papillary adenocarcinoma growing in the bile duct. The tumor cells have eosinophil granules, and the tumor was diagnosed as an oncocytic intraductal papillary neoplasm of the bile duct (IPNB), with invasion. (C,D) (×40) IPNB with a different form to that of the main tumor in the B4 periphery (C) and proximal side of segment 3 (D). (E) (×40), (F) (×200) The main tumor invaded the proximal side of segment 2. (G,H) (×200) Biliary intraepithelial neoplasia‐2/3 lesion is visible throughout the entire excised specimen

Occupational CCA consists of the mass‐forming type of intrahepatic CCA showing well‐, moderately or poorly differentiated adenocarcinoma , and the intraductal growth type of intrahepatic CCA and/or papillary‐type extrahepatic CCA, showing well‐differentiated papillary carcinoma (Figures 3 and 4). In the latter group, invasive cancerous portions were mucinous or tubular adenocarcinoma.^1^, ^4^, ^16^ Precancerous/preinvasive lesions, such as biliary intraepithelial neoplasia (BilIN) and intraductal papillary neoplasm of the bile duct (IPNB), were detected in various sites of the large intrahepatic bile ducts and/or hilar bile ducts and peribiliary glands (Figure 4). Chronic bile duct injury such as sclerosis of the bile duct with variable inflammatory cell proliferation, biliary epithelial injuries/focal bile duct loss, and biliary epithelial hyperplasia, were also observed in various sites of the bile ducts in the noncancerous hepatic tissues (Figure 4). When the diagnostic imaging and pathological findings were considered together, regional dilation of the bile ducts without tumor‐induced obstruction revealed pathological findings such as BilIN, IPNB, and/or chronic bile duct injury (Figure 5).^16^ Patients with occupational CCA had higher proportions of regional dilatation of the bile ducts without tumor‐induced obstruction and precancerous lesions (such as BilIN and IPNB) than did patients with non‐occupational CCA.^14^

**Figure 6:**
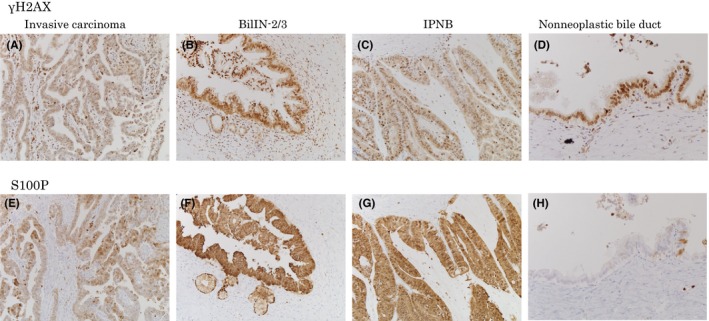
Immunohistochemical analysis for neoplastic changes and DNA injury.^16^ (A,E) Immunohistochemical expression of S100P and γ‐H2AX in invasive carcinoma. (B,F) Biliary intraepithelial neoplasia (BilIN)‐2/3. (C,G) Intraductal papillary neoplasm of the bile duct (IPNB) (×200). (D,H) In non‐neoplastic bile duct, although S100P expression is relatively weak or absent (H), γH2AX expression is observed (D)

Immunohistochemical analysis using primary antibodies against S100P and γH2AX to evaluate neoplastic changes and DNA injury gave highly positive results for the γH2AX and S100P markers in invasive carcinoma, BilIN, and IPNB; but positive γH2AX results and negative S100P results for non‐neoplastic biliary epithelium (Figure 6).^16^, ^17^ These results indicate that the carcinogenic process of occupational CCA comprised chronic bile duct injury and DNA damage in almost all the large bile ducts, along with induction of precancerous/preinvasive lesions and development of invasive carcinoma. Positive immunohistochemical expression of a theta‐class glutathione S‐transferase (GST) T1‐1 (a key enzyme for DCM metabolism) was observed in foci of BilIN, IPNB and CCA as well as in non‐neoplastic bile ducts.^17^ No cirrhotic changes or other hepatobiliary diseases were detected in the non‐cancerous hepatic tissues.

## 5 MOLECULAR BIOLOGICAL FINDINGS

Whole‐exome analyses of surgical specimens from four patients with occupational CCA showed an average of 44.8 somatic mutations per Mb in the genome of the CCA tissues; the frequency of somatic mutations was approximately 30‐fold higher than that of control common CCA tissues.^18^ Predominate mutations included C:G‐to‐T:A transitions with substantial strand bias, and unique trinucleotide mutational changes of GpCpY to GpTpY and NpCpY to NpTpY or NpApY in all of the occupational CCA genomes. Whole‐genome analysis of *Salmonella typhimurium* strain TA100 exposed to DCP revealed a partial recapitulation of the mutational signature seen in the occupational CCA. These findings indicate that such agents caused similar changes in the genome, and could be useful as a biomarker for occupational CCA.

## 6 TREATMENTS AND PROGNOSIS

Of the 18 patients from the printing company in Osaka, 13 underwent surgical resection; outcomes in 17 of the 18 patients were previously reported.^19^ Curative resection could not be carried out for four of the 13 patients because of extensive tumor invasion and the presence of precancerous/preinvasive lesions in the resected stump of the bile ducts. Of the 13 patients who underwent dissection or sampling of the lymph nodes, five patients exhibited metastases to the lymph nodes around the common bile duct or the common hepatic artery and/or peripancreatic lesions. Nine patients received adjuvant chemotherapy with fluorouracil, gemcitabine, and/or S‐1 (tegafur/gimeracil/oteracil potassium); two of the nine patients underwent radiation to the bile duct stumps. Among the 13 patients who underwent surgical treatment, intrahepatic recurrence or recurrence at the bile duct stump occurred in eight patients, and lymph node metastasis occurred in two patients. In four patients, solitary intrahepatic recurrence or solitary recurrence at the common bile duct distant from the primary carcinoma occurred; these recurrences might have had multicentric origins. Of the 13 surgical patients, four patients died of carcinoma and one patient died of hepatic failure with the progression of extensive hepatic fibrosis.^20^ In the remaining five of the 18 patients, chemotherapy or conservative treatment was given because of the advanced stage of the disease; these five patients died of advanced carcinoma.

Patients with occupational CCA seemed to have a high incidence of postoperative complications, including intra‐abdominal infection; this might be related to damage to the bile ducts, such as chronic bile duct injury and precancerous/preinvasive lesions. Postoperative recurrences from a multicentric origin might have occurred in some patients because DNA damage to the bile ducts greatly increased the potential for carcinogenesis. Aggressive treatment, including a second resection for such multicentric recurrences, appears to be effective because two patients are alive without recurrence after repeated resections of the recurrent tumors.

A recent immunohistochemical study of 10 tumors from nine patients with occupational CCA showed that the carcinoma cells expressed programmed death‐ligand 1 (PD‐L1) in all tumors and occasional PD‐L1 expression was observed in precancerous/preinvasive lesions such as BilIN and IPNB.^21^ These results imply that an immune checkpoint inhibitor may be a promising therapeutic option for patients with occupational CCA.

## 7 MECHANISM OF CARCINOGENESIS

In the printing company in Osaka, large amounts of chemicals, including chlorinated organic solvents, were used to clean ink residues. Epidemiological studies have suggested DCP and DCM to be causative agents.^1^, ^2^, ^5^, ^6^ Other chemicals used in this company were ruled out as possible causative agents because of their lower exposure, and/or shorter periods of exposure. In addition, other chemicals have been used in various types of industries without inducing cancer. However, identifying all components of chemicals previously used in the company is impossible because such chlorinated organic solvents were retired in October 2006, and the organic solvents included impurities.

In mammalian species, metabolism of DCM proceeds through cytochrome P450 (CYP) 2E1, which is abundant in the hepatocyte‐dependent oxidative pathway,^17^ producing carbon monoxide and GST T1‐1, which is abundant in the bile duct‐dependent pathway,^17^ resulting in the production of highly reactive intermediates, formaldehyde, carbon dioxide, and S‐(chloromethyl) glutathione, which promotes mutagenesis by forming guanine adducts. Amounts of DCM metabolized through the GST T1‐1 pathway increase at excess exposure to DCM; its products are strongly implicated in genotoxicity and carcinogenicity. However, the metabolic pathway and the mode of mutagenicity for DCP are still unclear.

Pathological studies found chronic bile duct injury with DNA damage, and precancerous/preinvasive lesions such as BilIN and IPNB in various sites of the bile ducts, along with invasive carcinoma. Whole‐exome analysis of the CCA tissues showed hypermutation, C:G‐to‐T:A transitions with substantial strand bias, and unique trinucleotide mutational changes. These findings indicate that chlorinated organic solvents or their products initiate a multistep carcinogenesis process on occupational CCA by causing DNA damage to the biliary epithelium, thus inducing chronic bile duct injury, precancerous/preinvasive lesions and, eventually, invasive CCA. Wide distribution of bile duct injury with DNA damage and precancerous/preinvasive lesions indicates highly malignant potential at the various sites of the bile duct which can lead to multifocal (multicentric) carcinogenesis.

However, detailed mechanism of carcinogenesis by chlorinated organic solvents is still unclear. In the experimental models using animals, exposure to such chemicals could not induce the CCA. Further investigations are necessary to elucidate the carcinogenic process.

## 8 POLICIES BY JAPANESE GOVERNMENT AND WHO

As described above, The Ministry of Health, Labour and Welfare of Japan classified CCA as a result of exposure to long‐term high concentrations of DCP and/or DCM as an “occupational disease.”^3^ Treatment cost for occupational CCA can be covered by compensation insurance, and workers who have been exposed to long‐term high concentrations of DCP can receive regular health examinations including laboratory tests and diagnostic imaging such as ultrasonography twice a year to detect occupational CCA. Based on the studies,^1^, ^2^ in June 2014, IARC reported that DCP was classified as a Group 1 carcinogen (carcinogenic to humans) and DCM was classified as a Group 2A carcinogen (probably carcinogenic to humans).^22^ The Japan Society for Occupational Health made similar classifications.^23^

## DISCLOSURE

Funding: This study was supported in part by Health and Labor Sciences Research Grants for Research on Occupational Safety and Health (epidemiological and cause‐investigated study of cholangiocarcinoma in printing company workers) and by Industrial Disease Clinical Research Grants (establishment of diagnostic methods for occupational cholangiocarcinoma; 14040101‐01). This work was also supported in part by the Japan Society for the Promotion of Science KAKENHI Grant Number 26462048 (clinicopathological and molecular biological analysis of carcinogenesis of intrahepatic cholangiocarcinoma by chemicals) and Grant Number 26462048 (Elucidation of carcinogenesis and establishment of treatments for intrahepatic cholangiocarcinoma by genetic and immunological analyses).

Conflicts of Interest: Authors declare no conflicts of interest for this article.
